# Children ASD Evaluation Through Joint Analysis of EEG and Eye-Tracking Recordings With Graph Convolution Network

**DOI:** 10.3389/fnhum.2021.651349

**Published:** 2021-05-25

**Authors:** Shasha Zhang, Dan Chen, Yunbo Tang, Lei Zhang

**Affiliations:** School of Computer Science, Wuhan University, Wuhan, China

**Keywords:** autism spectrum disorder, EEG, eye-tracking, multi-modality fusion, graph convolution network

## Abstract

Recent advances in neuroscience indicate that analysis of bio-signals such as rest state electroencephalogram (EEG) and eye-tracking data can provide more reliable evaluation of children autism spectrum disorder (ASD) than traditional methods of behavior measurement relying on scales do. However, the effectiveness of the new approaches still lags behind the increasing requirement in clinical or educational practices as the “bio-marker” information carried by the bio-signal of a single-modality is likely insufficient or distorted. This study proposes an approach to joint analysis of EEG and eye-tracking for children ASD evaluation. The approach focuses on deep fusion of the features in two modalities as no explicit correlations between the original bio-signals are available, which also limits the performance of existing methods along this direction. First, the synchronization measures, information entropy, and time-frequency features of the multi-channel EEG are derived. Then a random forest applies to the eye-tracking recordings of the same subjects to single out the most significant features. A graph convolutional network (GCN) model then naturally fuses the two group of features to differentiate the children with ASD from the typically developed (TD) subjects. Experiments have been carried out on the two types of the bio-signals collected from 42 children (21 ASD and 21 TD subjects, 3–6 years old). The results indicate that (1) the proposed approach can achieve an accuracy of 95% in ASD detection, and (2) strong correlations exist between the two bio-signals collected even asynchronously, in particular the EEG synchronization against the face related/joint attentions in terms of covariance.

## 1. Introduction

Autism spectrum disorder (ASD) is an early-onset neurodevelopmental disorder characterized by the impairments in social communication and repetitive behaviors (Lord et al., 2020), which severely affects the daily activities of children and occurs with an increasing trend year by year (Zwaigenbaum and Penner, 2018). In order to cater to the needs of the growing population of children with ASD, the early screening/assessment acts as a critical strategy to find effective solution to ensure that children with ASD and their families receive the imperative attention and achieve systematically optimal treatment process (Zwaigenbaum et al., 2015), e.g., behavior measurement (Bosl et al., 2018).

Recent advances in neuroscience indicate that analysis of bio-signals such as rest state electroencephalogram (EEG) and eye-tracking data can provide more reliable evaluation of children ASD than traditional methods of behavior measurement relying on scales do. Typically, EEG measurement has applied in monitoring atypical brain development and the extracted EEG features have acted as effective early biomarkers to distinguish children with ASD from those with typically developing (TD) (Bosl et al., 2011, 2018). Later, as the characteristics of children with ASD are mainly manifested in the impairment of directional attention and joint attention to eye gaze (Mundy and Newell, 2007), eye-tracking technology can well measure those behavioral changes in early attention in children with ASD (Wadhera and Kakkar, 2019), such as eye movements and responses to verbal and non-verbal cues (Duchowski, 2003).

In this paradigm, existing methods on children ASD evaluation through bio-signal analysis fall into two categories, i.e., *single-modality analysis* and *multi-modality fusion analysis*:

- Single-modality analysis directly operates on single data source (EEG or eye-tracking recordings). Specifically, EEG-based methods routinely apply power spectrum analysis (Coben et al., 2008), functional connectivity analysis (Peters et al., 2013), and those based on information theory (Hadoush et al., 2019; Zhang et al., 2020), while eye-tracking recordings are conventionally processed by statistical analysis (Fadi Thabtah, 2013) or machine learning-based methods (Carette et al., 2019) to characterize the critical biological ASD features. Then the extracted features are individually fed into classifier like support vector machines (SVM) (Bi et al., 2018) and decision tree (Thabtah and Peebles, 2020) for ASD detection. However, the effectiveness of the single-modality analysis may largely lag behind the increasing requirement in clinical or educational practices as the “bio-marker” information carried by the bio-signal of a single-modality is likely insufficient or distorted;
- Multi-modality fusion analysis aims to investigate bio-signals from multiple sources and theoretically enables to capture more abundant biological information to achieve the advantageous results over the single-modality counterpart (Zhang et al., 2021). Taking ASD evaluation via joint analysis of EEG & eye-tracking recordings for instance, studies have shown that eye-tracking recordings have strong correlation with EEG acquisitions making it possible for the comprehensive analysis between the behavioral characteristics (eye-tracking) and the brain dynamics (EEG) (Elison et al., 2013). Along this direction, existing methods mainly rely on feature-level fusion (Thapaliya et al., 2018) or decision-level fusion (Kang et al., 2020) to support the joint analysis. Nevertheless, these simple kinds of fusion strategies may not make full use of modality-cross information, thus the performances are largely limited.

To this end, grand challenge remains to explore the relationship between EEG and eye-tracking recordings and support the joint ASD detection with high performance.

To address the above issues, this study proposes an approach to joint analysis of EEG and eye-tracking for children ASD evaluation (section 3). The approach focuses on deep fusion of the features in two modalities as no explicit correlations between the original bio-signals are available, which also limits the performance of existing methods along this direction. First, the synchronization measures, information entropy, and time-frequency features of the multi-channel EEG are derived. Then, a random forest applies to the eye-tracking recordings of the same subjects to single out the most significant features. A graph convolutional network (GCN) model then naturally fuses the two group of features to differentiate the children with ASD from the typically developed (TD) subjects.

Experiments have been carried out on the two types of the bio-signals collected from 42 children (21 ASD and 21 TD subjects, 3–6 years old) (section 4) to evaluate the performance of the proposed approach in ASD detection and relationship discovery between the two bio-signals, e.g., EEG synchronization and the face-related/joint attentions.

To summarize, the main contributions of this study are as follows:

1. This study proposes a novel children ASD evaluation approach via joint analysis of EEG and eye-tracking recordings using graph convolution network with superior performance achieved. The solution holds potentials in the applications when concerning fusing much more data sources.
2. This study highlights the functional relationship between EEG and eye-tracking recordings, where strong correlation is discovered between the two bio-signals even collected asynchronously, especially for face-related/joint EEG synchronous attention covariance.

## 2. Related Work

This section introduces the most salient work closely related to this study from the prospective of ASD detection and exploratory relationship discovery between eye-tracking and EEG recordings.

### 2.1. ASD Detection

Existing methods concerning children ASD detection focus on (1) single-modality EEG analysis and (2) multi-modality EEG & eye-tracking fusion analysis. The most salient works along this direction are recapped as the follows.

#### 2.1.1. Single-Modality EEG Analysis

Kang et al. proposed a multi-feature fusion method using EEG for ASD detection (Kang et al., 2018). The method first computed power spectrum, bicoherence, entropy, and coherence features from EEG signals, then applied the minimum redundancy maximum correlation (mRMR) algorithm to choose to select the representative features, which are fed into SVM for classification. The results showed that the method could achieve 91.38% accuracy in ASD detection with only nine features.

In Thabtah and Peebles (2020), Thabtah et al. improved a novel Rules-Machine learning for ASD screening process along with offering knowledge bases to understand the latent ASD mechanism. Empirical results on children EEG indicated that Rules-Machine learning enabled to offer high classification performance superior to traditional approaches like boosting, bagging, and decision trees.

Wan et al. (2019) investigated the fixation times of 37 ASD and 37 TD children when watching a 10-s video of a female speaking. It was discovered that children with ASD showed significant reduction in fixation time at six areas of interest (AOI) and the discriminant analysis revealed that fixation times at the mouth and body could effectively discriminate ASD one from TD one with classification accuracy, sensitivity, and specificity of 85.1, 86.5, and 83.8%, respectively.

#### 2.1.2. Multi-Modality EEG and Eye-Tracking Fusion Analysis

Thapaliya et al. (2018) jointly analyzed EEG and eye-tracking recordings for children ASD detection, where SVM, deep neural network, logistic regression, and naive Bayes were, respectively, used for classification. The results presented that logistic regression obtained the highest performance than other classifiers and multi-modality analysis manifested superiority over the single-modality sone.

Later, Kang et al. (2020) developed a joint analysis framework to discriminate children with ASD from those with TD using two-modality data sources (EEG and eye-tracking). In the framework, power spectrum analysis was utilized to extract EEG features while the face gaze analysis was applied to characterize eye-tracking data, then the minimum redundancy maximum relevance method and SVM were, respectively, employed for feature selection and classification. The framework achieved accuracy of 0.85 and AUC of 0.93 in joint ASD detection.

### 2.2. Exploratory Relationship Discovery Between Eye-Tracking and EEG Recordings

Literatures have long discussed that there exists strong correlation between joint attention (JA) and neural activities in children with ASD. The most closely research is introduced as follows.

Studies in Elison et al. (2013) indicated a direct relationship between increased frontolimbic neural circuit connectivity at 6 months and subsequent Responding to JA (RJA) abilities at 9 months in TD individuals. Later, Billeci et al. integrated EEG with eye-tracking recordings to explore the visual patterns of RJA and the initiation of JA (IJA) (Billeci et al., 2017). It was found that 6-month treatment was accompanied by changes in eye-tracking measures partially correlated with the EEG features.

Vettori et al. used eye-tracking and EEG recordings with fast periodic visual stimulation to explore social communication difficulties in ASD (Vettori et al., 2020). The results illuminated that there was no interaction between group and stimulus category for simultaneously recorded eye-tracking data, but eye-tracking & EEG recordings were strongly correlated.

Lauttia et al. (2019) examined approach-motivation related brain activity (frontal EEG asymmetry) in response to direct and averted gaze in 3- to 6-year-old children with ASD, TD, or intellectual disability (ID). The study found that direct gaze elicited greater approach-related frontal EEG activity than did downcast gaze for children with TD. In addition, the response to eye contact in children might engage active-motivational brain systems and a pattern of EEG activity repeatedly connected with approach-related behavioral tendencies.

As a contrast to the above, this study focuses on the joint analysis of eye-tracking and EEG recordings with the following considerations: (1) measure the functional connectivity relationship between EEG and eye-tracking recordings, and (2) achieve multi-modality fusion between eye-tracking and EEG features based on Graph theory and support effective ASD detection task.

## 3. ASD Evaluation via Joint Analysis of Multi-Modality Recordings

**Figure 1:**
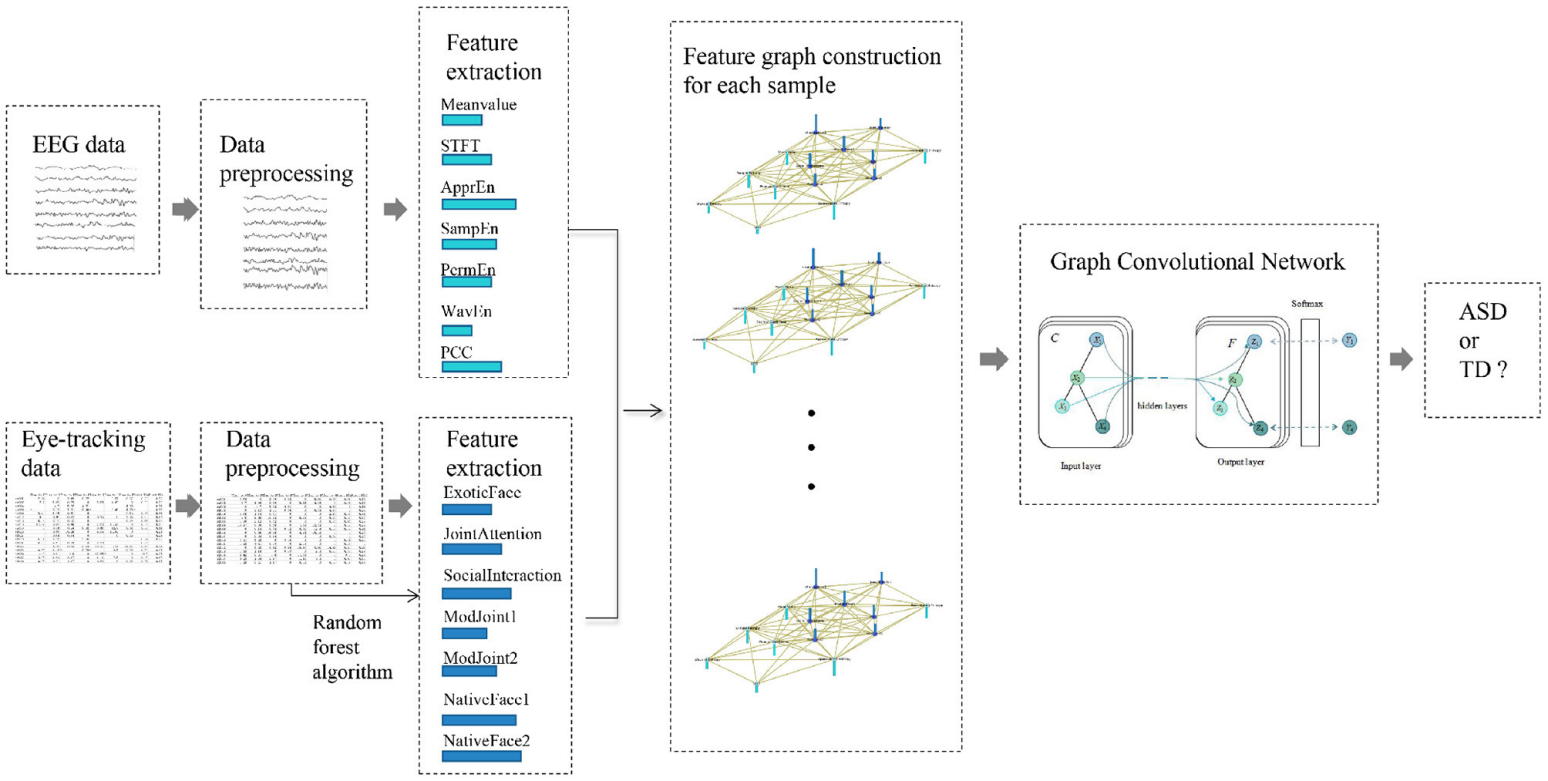
Overall design of the proposed method. The method mainly includes four parts: (1) data preprocessing for EEG data and eye-tracking data separately; (2) feature extraction; (3) feature graph construction for each sample; (4) graph convolution network for ASD evaluation.

This section details the overall design of the proposed method as depicted in Figure 1: (1) feature extraction of EEG and eye-tracking recordings, (2) feature graph construction, and (3) GCN with multi-modality feature fusion to discriminate children with ASD or TD.

### 3.1. Feature Extraction

Extracting the robust and discriminative features is among the most critical step for general bio-signal analysis (Chen et al., 2019). This study mainly includes the feature extraction of EEG and eye-tracking recordings.

Multi-domain features are captured to highlight the characteristics of EEG signals, e.g., information entropy, time-frequency analysis, and synchronization analysis. Specifically, information entropy features are first obtained by measuring the complexity of the original EEG signal individually along each channel, e.g., approximate entropy (ApprEn, Meedeniya et al., 2019), sample entropy (SampEn, Liu et al., 2017), and permutation entropy (PermEn, Kang et al., 2019). Then, the original EEG is processed by short-time Fourier transforms (STFT) to capture the time-frequency representation, e.g., entropy feature. In addition, in order to measure the functional connectivity between different brain regions, Pearson's correlation coefficient (PCC) is computed between each pair of EEG channels and later acts as the channel-domain feature.

Eye-tracking recording can provide more objective and accurate measurement of attentional patterns (e.g., measuring duration or latency of attentional engagement with a stimulus with millisecond-level precision), which is particularly suitable for those experiencing difficulties with following verbal instructions and handling complex social and cognitive demands, such as children with ASD. In this study, eye-tracking recording mainly includes testing the difference between children with ASD and those with TD in the observation of national or exotic faces, the difference in joint attention, whether there is gaze following, and difference in social interaction. Hence, the eye-tracking features include the time when, respectively, fixed on the face, eyes, body, and so on for the first time, which are revealed by the AOI (Kang et al., 2020). The time information like such as total fixation duration on certain AOI is recorded to quantify each child's engagement for each AOI, where a 60 ms threshold is applied to avoid counting unconscious gazing. Note that random forest algorithm (Meedeniya et al., 2019) is utilized to filter out the most significant features.

### 3.2. Feature Graph Construction

Inspired by the exploratory relationship discovery between eye-tracking and EEG recordings in section 2.2, this study considers to fuse EEG features and eye-tracking features and enable joint ASD analysis under graph theory.

Given EEG features and eye-tracking features of a sample, an undirected graph can be defined as G=(V,E,A), where V represents the set of nodes ($V={\left\{v_{i}\right\}}_{i=1}^{n}$, *n* is the number of nodes) and E is the set of edges connecting these nodes with connection relationship (adjacency matrix) denoted as *A* ∈ ℝ^*n* × *n*^ (Kipf and Welling, 2016).

#### 3.2.1. Nodes in the Feature Graph

The selected EEG features and eye-tracking features are defined as nodes of the graph (e.g., information entropy and total fixation duration on AOI) with corresponding attributes specified as the values of each group of features, denoted as $X={\left\{x_{i}\right\}}_{i=1}^{n}$. The number of nodes *n* in each feature graph is totally 14.

#### 3.2.2. Edges in the Feature Graph

The covariance between features is first utilized to measure the connectivity (edges) between nodes in graph. Then the binary adjacency matrix *A* is obtained by a certain threshold with covariance more than threshold set to 1 otherwise set to 0 [*A*(*i, j*) = 1: nodes *v*_*i*_, *v*_*j*_ are connected, *A*(*i, j*) = 0: nodes *v*_*i*_, *v*_*j*_ are unconnected].

**Figure 2:**
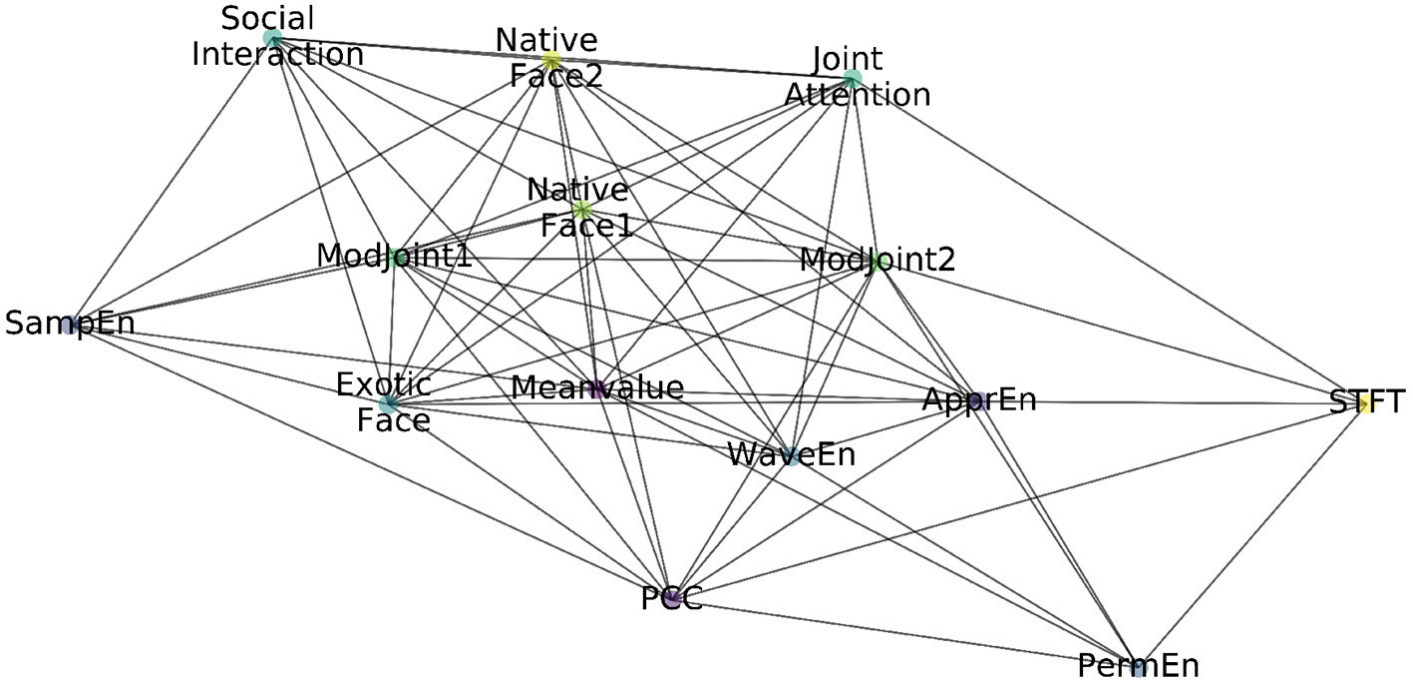
The constructed feature graph.

In this way, the constructed feature graph based on EEG & eye-tracking features is shown in Figure 2, including nodes, edges, and attribute values on each node.

### 3.3. GCN for ASD Detection

Graph network model has long been utilized for graph data processing with irregular connections between nodes in a graph (Kipf and Welling, 2016). The goal of this model is to learn a mapping function (linear or non-linear) from signals or features on a graph G=(V,E,A). Specifically, each graph convolutional layer can be written as follows:

(1) $$\begin{array}{r} H^{\left(l+1\right)}=f\left(H^{\left(l\right)},A\right) \end{array}$$

where *H*^(0)^ = *X* (input) and *H*^(*L*)^ = *Z* (graph-level output) with the number of layers *L*.

On the basis of the constructed feature graph in section 3.2, a GCN model with 2 hidden layers is built to perform ASD detection, where the features from multi-modality inputs are implicitly fused. Note that the original feature matrices *X* are concatenated sample by sample as $\hat{X}$ while a sparse block-diagonal matrix Â is obtained with each block corresponding to the adjacency matrix *A* of one graph.

In graph convolution operation, the block-diagonal matrix Â is first normalized as $\tilde{A}={\tilde{D}}^{-\frac{1}{2}}Â{\tilde{D}}^{-\frac{1}{2}}$, where $\tilde{D}$ denotes the diagonal degree matrix of Â. The GCN model then takes the simple form as follows:

(2) $$\begin{array}{r} Z=f\left(X,A\right)=softmax\left(\tilde{A}ReLU\left(\tilde{A}XW^{0}\right)W^{1}\right), \end{array}$$

where *W*^0^, *W*^1^ are weight matrices. The cross-entropy error acts as loss function, which is computed over all examples as:

(3) $$\begin{array}{r} L=-\underset{l\in y_{L}}{\sum }\overset{F}{\underset{f=1}{\sum }}Y_{lf}\ln Z_{lf} \end{array}$$

where *y*_*L*_ is the set of node indices that have labels and *F* denotes the number of feature maps.

**Figure 3:**
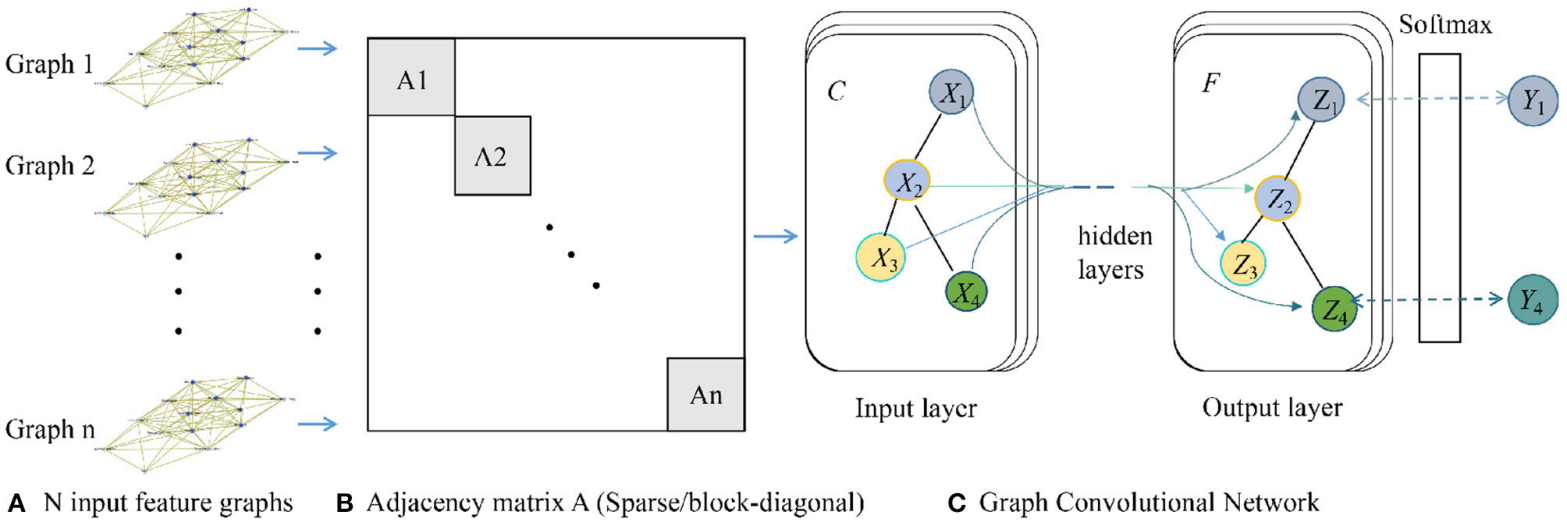
Architecture of our graph convolutional network (GCN) model.

In summary, the overall GCN model for ASD detection is shown in Figure 3.

## 4. Experiments and Results

To examine the effectiveness of the proposed approach for children ASD detection, experiments have been carried out to (1) evaluate the profit of introducing multiple modality (i.e., EEG and eye-tracking recordings) into the classification, and (2) compare the performance of the proposed method with the counterparts in ASD detection.

### 4.1. Data Source

The dataset used in the experiments consists of 42 subjects (21 ASD and 21 TD children) aged between 3 and 6 years. No statistical differences in age and gender are observed between ASD and TD groups. At each subject, EEG is recorded lasting for 6 min in a shielded room. A 128-channel HydroCel Sensor Net System (Electrical Geodesics, Inc.) was used for data recording. EEG recordings were re-referenced to an ear-linked reference. The EEG recordings are then filtered into the bands of [0.5, 45] Hz and sampled at 256 points per second. The ICA approach is adopted to remove the artifacts in EEG and visual inspection is performed to reject these segments contaminated seriously with noise. ICA decomposes observed signal into independent components (ICs). Once ICs are extracted from original signals, the clean signal reconstructed by discarding ICs contained artifacts such as muscle activity, eye movement, and blink artifacts. With the aid of EEGLAB toolboxes that support ICA-based artifact removal from EEG, the artifact-corrected EEG can be achieved. In order to reduce computational overhead, 8 electrodes (F3,F4,T3,C3,C4,T4,O1,O2) were selected for analysis. The signals are then divided into segments with length of 4 s and no overlaps. The eye-tracking recording is recorded after the EEG recording process by performing about 7 eye-tracking tests for the subjects with sampling rate of 300 Hz. In this dataset, eye-tracking data were recorded for 7 tests designed for different purposes. The purpose of these different experiments mainly includes testing the differences between children with autism and normal children in the observation of national or exotic faces (“Nativeface1,” “Nativeface2,” and “Exoticface”), testing the differences in joint attention (“Joint Attention”), whether there is gaze following (“ModJoint1” and “ModJoint2”), and differences in social interaction (“SocialInteraction”). Before the formal experiment, a five-point calibration program is performed and the experiment proceeded after all 5 points are captured with small error vectors. The children are presented with a series of photos in sequence. Each type of photo appears 6 times and lasts for 10 s each time. To explore the child's engagement with each AOI, cumulative fixation duration within the selected AOI, defined as the time spent on that AOI, is analyzed, on which a threshold of 60 ms is applied to rule out the invalid value caused by unconscious looking.

### 4.2. Effectiveness of Multi-Modal Information

To examine the effectiveness of introducing multi-modal data for autism assessment, a number of experiments have been performed to compare the proposed method with the methods based on single modality. The baselines are constructed to, respectively, process EEG or eye-tracking data. T-test is first performed on the features of single modality to check the difference between ASD and TD. It should be noted that since the features used in the proposed method are consistent with the baselines, their T-test results are also the same. The *p*-value calculated shows that there existed significant difference between these two groups of subjects against most of features. The results of T-test are detailed in Table 1.

**Table 1:** T-tests t-statistic values and corresponding *p*-value each feature.

**Features**	**T-test results (statistic/*p*-value)**
Mean value	−22.1978/*p* < 0.001
STFT	3.7538/*p* < 0.001
Pearson correlation coefficient	−1.5973/*p* < 0.5
Approximate entropy	−6.6065/*p* < 0.001
Sample entropy	−2.6144/*p* < 0.01
Permutation entropy	0.1979/*p* < 1
Wavelet entropy	−3.8290/*p* < 0.001
Exotic strange face	−2.4775/*p* < 0.05
Social interaction	−2.8989/*p* < 0.01
Joint attention	−0.7883/*p* < 0.5
Mod joint1	−2.3232/*p* < 0.05
Mod joint2	−1.6238/*p* < 0.5
Native strange face1	−3.1576/*p* < 0.005
Native strange face2	−4.7105/*p* < 0.001

**Figure 4:**
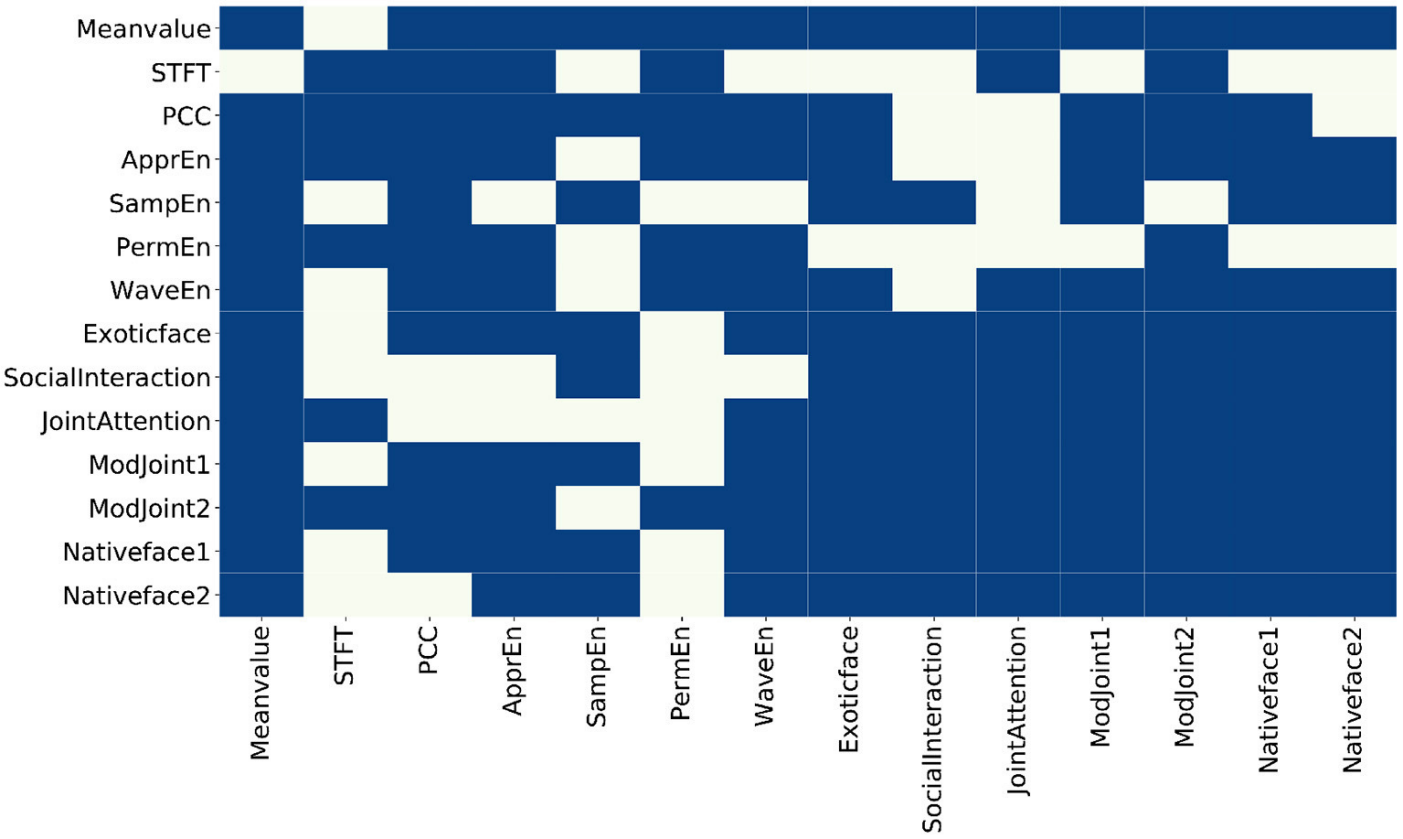
The constructed adjacency matrix. The blue block means there is a connection between two nodes, and the beige block means there is no connection between the two nodes.

From the results of each feature in Table 1, it can be found that (1) among the EEG features, the mean value feature (time-domain feature), the approximate entropy feature, and the wavelet entropy feature have the smallest *P*-value. This means that these three features differ the most in children with ASD and TD, and (2) among the eye-tracking features, ASD children and TD children have the largest difference in the feature of strange faces in their home countries, which coincides with previous studies. Second, there are obvious differences in social interaction between ASD and TD children, which is consistent with the existing researches, that is, ASD is characterized by difficulties in social communication and social interaction as well as repetitive behaviors and restricted interests (Georgescu et al., 2019). The proposed method used the adjacency matrix (see Figure 4) to input of network structure and perform GCN model training, where the ratio of training set against test samples is 8:2. The experimental results are shown in Table 2.

**Table 2:** Comparison between the proposed method against the methods based on electroencephalogram (EEG) or eye-tracking data.

**Evaluation metrics**	**Accuracy**	**Precision**	**Recall**	***F*_**1**_**
Method using EEG recording	0.7368	0.7500	0.9231	0.8276
Method using Eye-tracking data	0.7895	0.8000	0.9230	0.8571
Proposed method	0.9500	0.9500	0.9500	0.9500

As shown in Table 2, the results based on multi-modality data for autism assessment are significantly better than the results based on single modality. The more interesting result is that the results based on eye-tracking recording for autism assessment are better than those based on EEG recording. There are two possible reasons: On the one hand, EEG recording records the inner complex neuron activity, while the eye tracking data records the outer behavior, so it can portray the behavioral characteristics of children with ASD and TD more intuitively; On the other hand, the feature maps corresponding to the two types of multi-modality data are different, and the feature maps corresponding to the eye tracking recoding have more connections, so that more information can be used in model training.

### 4.3. Evaluation of Overall Performance

Two methods are introduced here to evaluate the overall performance of the proposed method. The structures are detailed as follows.

Kang et al. proposes a framework based on feature engineering for ASD detection (Kang et al., 2018). Features are extracted from EEG and eye-tracking recordings separately. Specifically, EEG features are collected by computing relative power of multiple sub-bands including delta (1–4 Hz), theta (4–8 Hz), alpha (8–13 Hz), beta (13–30 Hz), and gamma (30–45 Hz) bands over all electrodes are calculated. And for eye-tracking recording, eight AOI are selected for face photo analysis consisting of background, body, face, eyes, right eye, left eye, mouth, and nose. Analysis index is computed to quantify the child's engagement for each AOI, which is defined as the percentage of fixation time in the AOI vs. the total fixation time (fixation time in AOI/total fixation time). The minimum-redundancy-maximum-relevance (MRMR) method is then utilized to select proper features. SVM is employed for final classification.

**Figure 5:**
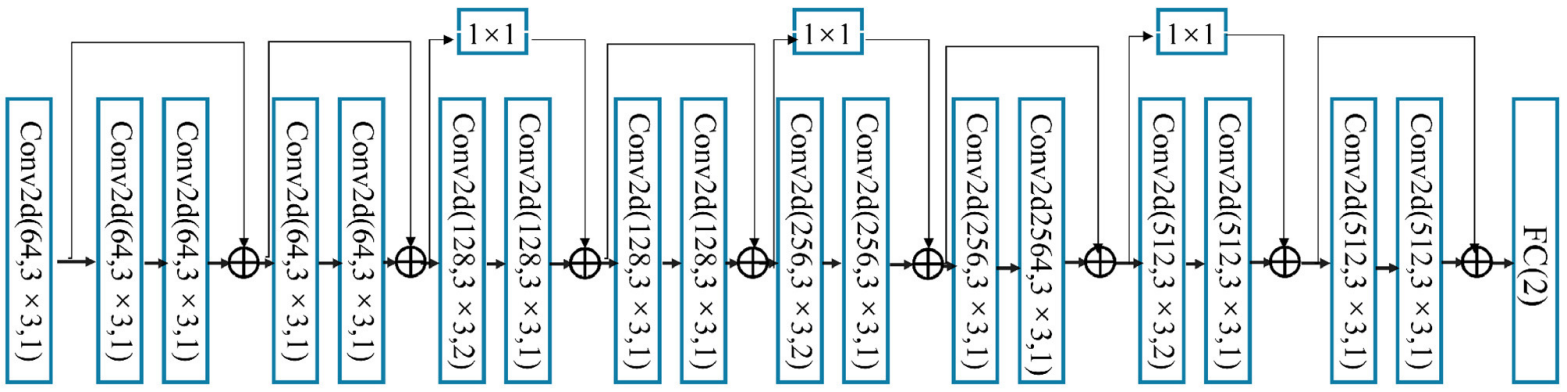
The structure of ResNet18 model.

Deep residual networks, or ResNets for short, are presented to alleviate the problem that deeper neural networks are more difficult to train. In this study, ResNet18 is introduced to classify EEG and eye movement data. At first, features from EEG and eye-tracking data are cascaded to perform feature-level fusion. Subsequently, the fused features are sent to ResNet18 for the classification of autism. Specifically, the ResNet18 contains 8 Res blocks, where the number of channels is configured as 64-64-128-128-256-256-512-512. Each Res Block mainly contains two convolutional layers, so the total number of convolutional layers is 8*2 = 16. A residual module of skip connection is added between every two neighboring blocks. Finally, the fully connected layer are following to perform the final classification. The overall architecture of ResNet18 is shown in Figure 5.

#### 4.3.1. Analysis of T-Test Results

It should be noted that the proposed method and the method based on ResNet18 have the consistent T-test results as they shares the same features. The resultant *p*-value shows that there exists significant difference between ASD and TD for most of the selected features. The results of T-test are specifically presented in Table 1. Similar results can be observed from T-test in Kang et al. (2018), which are shown in Table 3. Among EEG features, the *p*-value of theta and beta band are the smallest, that is, children with ASD and TD have greater difference in these two bands. The results are conforming to previous studies, which demonstrates a reduced increase of theta power (Yeung et al., 2016) and stronger beta band (Buard et al., 2018) in ASD children. Among the eye-tracking features, children with ASD and TD had the largest difference in the two characteristics of eyes and body, which is also consistent with the existing research (Falck-Ytter et al., 2013).

**Table 3:** T-tests t-statistic values and corresponding *p*-value each feature.

**Features**	**T-test results(Statistic/*p*-value)**
Delta (1–4 Hz)	2.0912/*p* < 0.05
Theta (4–8 Hz)	5.9232/*p* < 0.001
Alpha (8–13 Hz)	−2.1672/*p* < 0.05
Beta (13–30 Hz)	−6.1456/*p* < 0.001
Gamma (30–45 Hz)	1.8713/*p* < 0.1
Background	2.0025/*p* < 0.05
Body	2.4132/*p* < 0.05
Face	1.2096/*p* < 0.5
Eyes	1.8219/*p* < 0.1
Right eye	1.3180/*p* < 0.5
Left eye	2.2252/*p* < 0.05
Mouth	1.8355/*p* < 0.1
Nose	2.0168/*p* < 0.05

#### 4.3.2. Comparison of Overall Performance

Table 4 gives the comparison between the proposed method with the counterparts discussed above. From the result, it turns out that the proposed method is superior to Kang et al. (2018) algorithm in terms of diagnostic performance. By analyzing the details of the two methods, it can be found that the proposed method cannot only find the feature relationships between and within different modalities but also use these relationships to fuse features from different modalities. Finally, the proposed method makes good use of the complementary or supportive information of different modalities, and achieves a higher classification accuracy (95.00%), while Kang et al. (2018) simply concatenates the features of different modalities. In addition, the proposed approach achieves the highest classification precision (0.9500) compared with the ResNet18-based methods (0.8050). Although ResNet18 structure has a deeper level, it cannot achieve better diagnosis performance from the experiment. The possible reason is that simply and rudely cascading EEG and eye-tracking recordings cannot make good use of multi-modality information.

**Table 4:** Experimental results of our method and the compared methods.

**Evaluation metrics**	**Accuracy**	**Precision**	**Recall**	***F*_**1**_**
Our method	0.9500	0.9500	0.9500	0.9500
Kang et al., 2018	0.8100	0.8100	0.8100	0.8100
ResNet18	0.7000	0.8050	0.7150	0.6800

### 4.4. The Explanation of Feature Graph Edges

In probability theory and statistics, covariance is used to measure the joint change degree of two random variables. By calculating the covariance between every two features and performing threshold processing, the adjacency matrix of the feature map is obtained, and the visualization is shown in Figure 4.

The following information can be inferred from the adjacency matrix:

- Within EEG features, not every feature has a correlation with all of other EEG features. Specifically, mean value is related to other features except STFT. In addition, STFT is independent to sample entropy and wavelet since entropy based features are calculated to measure the complexity of time series while STFT is a localized process in time and frequency domains. Approximate entropy has nothing to do with sample entropy. Approximate and sample entropy are both important indicators to quantify the complexity of time series, but sample entropy had relative consistency with respect to approximate entropy. The sample entropy is also not related to sorting and wavelet entropy. However, in the modal of eye tracking, features are related to each other from the results.
- Between the two modes, mean value from EEG is related to all other eye-tracking features, which can be interpreted as the mean value of EEG related to the fluctuation of eye-tracking data; STFT is related to the 10th and 12th feature because both of them represent joint attention features. Synchronization feature is related to exotic face, the 11th and 12th features (joint attention, whether there was gaze following), and the 13th feature (native strange face 1). Sample entropy is related to the 8, 9, 11, 13, and 14th features. That is, entropy-based features are related to face features, joint attention, and social interaction features. Sorting entropy is related to joint attention. Finally, wavelet entropy is related to all other eye-tracking features except social interaction.

From the above analysis, it can be seen that, the way of representing the feature as the node and the covariance among features as the edges can provide a richer intra- and inter-modality information, so as to perform multi-modality fusion better.

## 5. Conclusions

Aiming at the grand challenges for reliable relationship discovery between multi-modality data sources and the joint children ASD auxiliary detection, this study proposes an approach to joint analysis of EEG and eye-tracking for children ASD evaluation. The approach focuses on deep fusion of the features in two modalities as no explicit correlations between the original bio-signals are available, which also limits the performance of existing methods along this direction. First, the synchronization measures, information entropy, and time-frequency features of the multi-channel EEG are derived. Then a random forest applies to the eye-tracking recordings of the same subjects to single out the most significant features. GCN model then naturally fuses the two group of features to differentiate the children with ASD from the TD subjects.

Experimental results indicate that (1) the proposed approach can achieve an accuracy of 95% in ASD detection, and (2) strong correlations exist between the two bio-signals collected even asynchronously, in particular the EEG synchronization against the face related/joint attentions in terms of covariance. In conclusion, the solution holds potentials in the applications when concerning fusing much more data sources.

## Data Availability Statement

The original contributions presented in the study are included in the article/supplementary material, further inquiries can be directed to the corresponding author/s.

## Ethics Statement

The studies involving human participants were reviewed and approved by The present study was conducted according to the Declaration of Helsinki and approved by the ethics committee of Beijing Normal University. Written informed consent to participate in this study was provided by the participants' legal guardian/next of kin.

## Author Contributions

SZ and DC designed the study. SZ performed the experiments and results analysis. All authors contributed to the writing of the manuscript.

## Conflict of Interest

The authors declare that the research was conducted in the absence of any commercial or financial relationships that could be construed as a potential conflict of interest.
